# The contribution of the flipped learning environment to value perception and controllability of classroom activities as antecedents of learners’ anxiety: A control-value approach

**DOI:** 10.3389/fpsyg.2022.1000710

**Published:** 2022-09-07

**Authors:** Li Liu

**Affiliations:** School of Foreign Studies, Zhongyuan University of Technology, Zhengzhou, China

**Keywords:** learners’ anxiety, controllability, value appraisal, flipped learning environment, control-value theory

## Abstract

Students enrolled in tertiary education encounter multiple challenges, which prevent them from being proficient. One of these challenges is anxiety which is a common achievement emotion that impacts many students. Anxiety may prevent learning and may be negatively related to learning due to the negative values of classroom activities and their low controllability. As a result, obtaining more research evidence on anxiety plays an important role in allowing learners to develop the skills they need in different types of technology-based environments such as Flipped Learning (FL). With the prevalence of Internet usage, FL is gaining increasing popularity among higher education individuals. The FL approach is an important model for modifying teaching, cultivating enthusiasm, and interaction, and developing educational presentations in student-focused learning circumstances. The potential affordances of the FL environment might place learners in more positive states of control and value appraisals than the environment of conventional classes, which can lead to the removal of negative emotions such as anxiety. Given the benefits of FL and the potential affordances of its environment, the purpose of this conceptual study is to argue how the inherent affordances of the FL environment can contribute to the controllability and positive values of classroom activities reducing learners’ anxiety in light of control-value theory.

## Introduction

In recent years, the quick evolutions of cellphone technology, the emergence of Web 2.0 sites, as well as social media growth led to an increased usage of IT in class to provide the requirements of learners in the twenty-first century (Andujar et al., 2020). The growing trend of online learning along with the corresponding changes in the educational context, which involves the introduction of new digital learning platforms, have driven many educators to identify the ways in which learners experience and create new learning opportunities (Martin et al., 2020). Today, due to the emergence of the 4th industrial revolution and its focus on the application of technology, one cannot draw the lines between the physical domain and digital domain of education. In the same vein, educational scholars have been examining the technological and human features of online learning, which influence the extent to which online learning is effective from a geological perspective (Weegar and Pacis, 2012; Rajabi, 2015; Aghaei and Gouglani, 2016; Aghaei et al., 2022).

A variety of factors are involved in learners’ success; more specifically, the learners’ achievement in classes depends on both cognitive elements and affective elements (Turner and Lindsay, 2003). Emotion is an aspect of educational settings, where the extent to which students are ready to participate in learning activities is influenced by affective aspects (Pekrun and Linnenbrink-garcia, 2012). Also, emotions are deemed as subjective experiences influenced by the context; therefore, it depends on the context. The literature refers to multiple emotions figured in research, such as disappointment, excitement, anxiety, eagerness, hope, and despair, among others (Goetz et al., 2010). One of the basic affections and a multi-aspect response to real or possible hazards is anxiety, which mixes physical, cognitive, affective, and behavioral elements indicating developing mechanisms to stay alive or handle the threatening provocations (Hohoff, 2009). For example, anxiety is an influential factor that affects Students’ achievement, impacting their short-term memory ability negatively. It can also act as a barrier to the Students’ knowledge development. Anxious learners are obsessed with how other learners judge them and what impression their peers have of them. These students find a learning situation very upsetting and are forced to give up the learning activity as they deem learning very unsettling (Çoruk and Çakır, 2017). Nowadays, teachers face a serious problem, namely, the inefficacy of traditional teaching methods. Indeed, these methods and strategies do not meet the new standards anymore. Today, learners need to gain a more comprehensive grasp of content since such understanding enables them to gain a better mastery of essential math skills. Indeed, the traditional instruction is not conducive to the interaction among the learners as it focuses on student-teacher interactions, which makes the learners struggle with the content (Deliktas and Stojkovska, 2019). One solution to address this problem is flipped classroom which is considered a type of blended learning since it is consistent with the new learning expectations. The flipped class is a modern concept of education that has attracted numerous schools and teachers’ attention in the recent 20 years. Thanks to flipped classrooms, teachers have a chance to build on several instructional strategies to deal with the skills required in this century (Teo et al., 2022). These skills play an essential role in tailoring the education to each individual’s needs (individualized learning) in a learner-centered context. In this context, active learning can be achieved, interaction is enhanced, and class time is used efficiently (Awidi and Paynter, 2019). The flipped class is aimed at encouraging learners to participate in the educational process and educational activities following their skills and interests (Van Alten et al., 2019). The philosophy of hypothesis behind the flipped class is that learners will get information out of the class, therefore, they can understand it in the class by getting involved in tasks that enhance high-order sophisticated contemplation and the ability to solve problems. Also, one possible benefit of the internet-based setting is improving experiences of learning and enhancing learners’ communication ability by mitigating Students’ anxiety (Ataiefar and Sadighi, 2017). Based on the literature, one can use both the internet and technology to limit anxiety in learning (Ataiefar and Sadighi, 2017). Therefore, educators can control Students’ anxiety experiences within the classroom through flexible and enjoyable flipped methods such as competitions, games, and activities related to problem-solving (Zarrinfard et al., 2021).

Using technology to accomplish the class for a learner needs an incentive, beyond which there is persistence and love that alludes to grit. Managers and academics hold that learners’ success prerequisites higher discipline and higher internal encouragement compared to a conventional class (Mosanya, 2019). Moreover, grit constructively affects psychological wellbeing through the relationship with low tension, dejection, anxiety, and enhanced constructive feeling (Datu and Restubog, 2020). Positive psychology (PP) is an extensive scientific field concentrating on investigating various elements that improve health with grit as a core concept (Seligman and Csikszentmihalyi, 2000). When positive psychology emerged, numerous positive psychologists took into account Students’ power to enhance their learning results (Wang et al., 2021). Indeed, based on research findings, non-cognitive skills and personality play an essential role in living a successful life, with grit found to be important in academic success. Perseverance of effort (PE) and consistency of interest (CI), as two aspects of grit, play an important role in an individual’s accomplishment, since perseverance leads to the achievement of mastery despite challenges or failure. CI is pivotal for keeping on deliberate practice to gain mastery (Datu, 2017). Grit differs from other such individual constructs due to its concentration on long-term psychological endurance, tolerance, flexibility, and resoluteness, and in various conditions; however, it is not an easily available target that permits quick feedback (Reed et al., 2013). Online learning importantly affects grit in technological teaching, improves Students’ involvement in online courses, and enhances Students’ success (McClendon et al., 2019).

According to some researchers, learners’ grit is reinforced by their engagement in blended learning (Cheung and Hew, 2011). Professors need to serve as a model of perseverance, confidence, and a positive attitude. Accordingly, they should combine online learning with in-person activities (Cassidy, 2015). The level of grit has proved to be very significant since it involves the persistence of learners’ interests and efforts. In consequence, the traditional teaching methods are characterized by a teacher-fronted aspect, undermining the Students’ self-confidence and determination. On the contrary, exciting online activities enhance the Students’ grit as these tasks are very challenging. Even though many educators and scholars have debated the positive effects of flipped learning (FL) (McLaughlin et al., 2016; Chen Hsieh et al., 2017; Hung, 2017), no study, seemingly, has examined the contribution of flipped classrooms to reducing learners’ anxiety. Given the moderating role of grit concerning anxiety, it can be investigated in more detail. Overall, flipped instruction contributes to the expansion of learners’ knowledge so that this expansion also occurs outside the classroom, providing learners with a variety of interactive collaborative and cooperative activities. These outcomes are mainly due to the inverted teaching process involved in flipped instruction. Consequently, one can view the such type of instruction as an appropriate replacement for the conventional approach, which helps to decrease Students’ anxiety. Yet, very few studies have dealt with this domain despite its importance. As suggested by Loderer et al. (2020), the promotion of Students’ achievement emotions and performance should show draw the attention of researchers in their future endeavors in light of the advances in technology-based learning. That is, the interaction between technology-based environments and achievement emotions can provide us with how control and value appraisal as the antecedents of these emotions can be realized in these environments. Thus, this conceptual review aimed to discuss how the FL environment, based on the literature on FL, can contribute to positive values and control appraisals of classroom tasks as two predictors of anxiety, as an achievement emotion.

## Review of the literature

### Anxiety

As a multi-faceted construct, anxiety is experienced by those who aim to learn so that they become anxious when they want to take part in learning and/or utilization (Horwitz, 2017). All humans experience anxiety as a normal feeling, which may be caused by multiple factors, including internal and external changes, novel circumstances, or perceived vagueness. In other words, the majority of people naturally feel anxious and experience pressure. This perceived pressure is considered anxiety, which occurs when the individual finds himself/herself in an unfamiliar situation (Cubukcu, 2013). In the same vein, anxiety has also been characterized as an excessive sense of concern and agitation, which is accompanied by physiological changes, the inability to predict reality and the quintessence of danger, and a lack of self-confidence regarding one’s ability to manage it (Hewitt, 2011). Anxiety has been defined as experiencing fear or insomnia after predicting danger, which brings about stress and uncertainty in the anticipation of potential worries (Yükselir and Harputlu, 2014). Overall, many factors influence anxiety (e.g., internal psychological moods, cognitive and emotive factors, contextual requirements, and the existence of others) (MacIntyre, 2017).

A review of the literature shows that anxiety is considered an intrinsic feeling and a social structure. Put it another way, anxiety is experienced due to factors, such as cognitive state, mental conditions, and affective moods along with a perceived pressure to learn (MacIntyre, 2017). This creates inconvenience thereby learners feel uncomfortable as they have no linguistic means at their disposal to authentically express themselves. In the view of Aydin (2013), anxiety is concerned with an agitating affective mood through which an individual feels threatened, experiences weakness, and feels stress in the face of anticipated danger. Overall, anxiety sets in when an individual faces inevitable conditions, which are sensed as physical or psychological threats (Szyszka, 2017). Anxiety, which unfolds as an individual’s natural response, is described as a troublesome emotion that is experienced in the anticipation of future risk. This leads to muscular tension and heightened alertness. This feeling is accompanied by some physiological, behavioral, and intellectual changes, which allow the person to protect himself/herself against potential risks to their honesty (Pinto et al., 2015). It should be noted that the FL environment makes learners’ anxiety sensitive to the affordances of this environment. This sensitivity to these affordances renders learners’ anxiety a dynamic emotion. It seems that due to the innovative nature of the FL environment or lack of familiarity with the inverted structure of this environment, some learners might find it anxiety-provoking.

### Flipped classroom

A review of the literature shows that FL found its way into higher education for the first time by the late 1990s, to allow for more active and diverse lessons for learners. Moreover, it was aimed at increasing the application of modern technologies to make the learning process more effective (Låg and Sæle, 2019). The conventional approaches are highly educator-oriented and students typically listen to the educator during class. It varies in FL and the time spent in the class includes enriched learning tasks, allowing students to more actively playing their roles within the class compared to the conventional approaches. FL refers to a learning method where learners learn the primary information by watching short clips within their homes and get to the class setting to comprehend their problems in learning and solve misconceptions (Murray et al., 2015). In the same vein, FL is described as a model of learning where the learners see relevant clips prior to the class and use class time to learn sophisticated problems, solve problems, and get connected to daily life conditions (Stone, 2012).

FL is defined as a mixed academic model allowing learner-oriented learning within the class through moving educator-instructed learning out of the class setting *via* affordances (Gopalan and Klann, 2017). Affordance refers to an action possibility shaped by the interaction between an agent and its surrounded environment (Gibson, 1977). Based on this definition, affordance in this study refers to the properties of the objects in the FL environment which enhance learners’ value perceptions and controllability of classroom activities. For instance, as an affordance, the clips act as a way of transmitting the course material to the learners as well as a means of digital learning (Knight, 2016). These affordances can enhance learners’ agency in terms of their sphere of influence. That is, thanks to learners’ engagement in classroom activities *via* affordances of the flipped environment, learners can enact their sense of agency by enhancing their sphere of influence on the anxiety-inducing factors in this environment.

The FL originates from the learning theory of constructivism and in the constructivist view, information is actively built by the students, rather than passively from the outer world. Learning takes place by the student, rather than being imposed on them (Strayer, 2012). Tasks before the class should extend beyond the internet and clips, identical goals can be gained through proper learning content and instruction (Kim et al., 2014). In this case, one definition provided for FL refers to it as a model of learning where learners learn the class content through written documents, clips, presenting, etc. taking advantage of the pre-class technological chances, and in-depth learning within the class through discussing, asking and answering questions and practical tasks by reinforcing, questioning and applying the underlying knowledge. As a model, FL involves direct teaching occurring outside of class mainly *via* videos. An in-class FL is aimed at providing the learners with more time for engaging in discussions about the topic, problem-solving activities, collaboration among the students, and individualized teaching activities tailored to individual needs (Francl, 2014; Shahnama et al., 2021).

In the view of Bergmann and Sams (2012), this method involves both video courses, as well as the meaningful and interactive activities, which are carried out in the class. Furthermore, FL is characterized by its contribution to increasing individual responsibility regarding learning (Fautch, 2015). In this active learning context, learners acquire the information they need regarding the course, while during the in-class time, they are provided with ample opportunities for collaborative and student-active activities. FL enhances learner-learner, student content, and learner-teacher interaction chance through online collaboration instruments, allowing learners to engage in more time in class (Akçayır and Akçayır, 2018). Parvaneh et al. (2022) carried out a study to examine the short and long-term impact of FL and language proficiency on autonomy and anxiety among English learners in Iran. This investigation showed that FL significantly influenced autonomy and anxiety. The difference in the nature of affordances in the online environment with that of a traditional classroom is related to the inverted structure of the flipped environment. The inverted structure of the FL environment provides unique affordances for the reduction of learners’ anxiety. Since learners receive instruction outside the class time, compared to traditional classes, they have much more time to reflect on the feedback they receive for their classroom activities inside the class, which improves their positive values for these activities. Also, this inverted structure keeps learners in control of the classroom activities because they have enough time to get involved in these activities and overcome issues related to these activities before class time.

### Arguments for the contribution of the flipped learning environment to the reduction of anxiety in light of the control-value theory

Control-value theory posits that achievement emotions are susceptible to the control and value appraisals of classroom activities. When learners feel negative and out of control of their assigned tasks, they feel negative emotions like anxiety (Pekrun, 2006). Learners’ anxiety in the FL environment can be reduced subject to the contribution of this environment to both the learners’ value perception of classroom tasks and their controllability of the classroom activities (see Shao et al., 2019). First, learners might find themselves in control of the classroom activities due to the potential affordances of the FL environment. When learners enter the class, they have already gained enough information about the topics *via* the content provided for them out of the class time. This high controllability is postulated as learners have more time than the conventional classes to watch the digital content and tasks assigned to them in the flipped environment. Furthermore, they can watch the videos and follow the related activities of these videos with more concentration as they are not pressed for time as they are in conventional classes. More specifically, the flexible timing for doing classroom activities is a potential affordance of a FL environment which can remove the negative feeling of anxiety induced as a negative filter while doing content and material-related tasks in this environment. As a result of this removal of the negative filter (see Krashen, 1982), learners can find themselves quite competent in doing the assigned tasks. This is quite in line with the broaden-and-build theory of positive emotions (Fredrickson, 2001), indicating that the removal of negative filters such as anxiety enhances learners’ attentional scope and heightens their concentration. Thus, the flexible timing of FL can help learners carry out class activities without any cognitive demand due to the time pressure which frequently occurs in conventional classes. Therefore, they will make more consistent efforts and maintain their perseverance for the achievement of their long-term goals. Also, feeling focused and competent in their assigned tasks, language learners experience moments of low level of anxiety in the FL environment.

On the other hand, the significant contribution of FL environments to positive values of classroom tasks should not be neglected. Learners in this environment can find how well the activities can be tailored to their individualized needs. Also, they can find more space for interaction with their classmates without the fear and anxiety of being judged by their teacher in this environment. Besides, due to the contagious nature of emotions (see Shao and Parkinson, 2021) learners’ positive emotions in the flipped environment can be reciprocated. Another reason for the positive values of the activities and tasks in the FL environment can be the affective and cognitive preparation prior to the beginning of the class sessions. Before the class, learners have the chance to learn the taught contents and videos and take advantage of their cognitive strategies to cope with the challenges they face while doing the related activities without time pressure. This mental readiness broadens their mental capacities and positions them in a positive affective state during class time when they can discuss their issues and problems with their teacher and classmates. This means that the positive perception they achieve prior to the in-class discussion can also help learners improve their reflection and metacognitive strategies during the class sessions. It should be mentioned that the affordances embedded in the FL environment should also be viewed as mediators *via* which individual learners can internalize their experiences in the flipped environment until they fully develop their skills and achieve independence in their application in the classroom environment. This process can help them reduce their anxiety as they gain more mastery experience within the FL environment.

## Conclusion, implications, and future directions

Fortunately, stress and anxiety can be alleviated through the effective application of teaching methods as implementing technologies encourage the learners to engage more actively in the learning activities that can also increase their motivation and achievement. Indeed, FL highlights learner readiness before class and concentrates on active student-oriented learning; therefore, it is useful for EFL students and permits them to decrease their anxiety within the classroom (Goda et al., 2017). Based on the literature review, Students’ anxiety in FL comprises the type of anxiety in the process of learning and their successes as well as communal anxiety, regarding the fear to establish communication and/or cooperation with people. As one of the most effective predictors of educational success in today’s education, grit plays an essential role in enhancing learners’ capacity to persist and accomplish their long-term goals. It also helps the learners to persevere even in the face of hardship, challenges, or adverse conditions throughout schooling and life. As stated by Lan and Moscardino (2019), grit is a determining predictor of educational success in a COVID-19 pandemic in which most process of learning in this era is online. As a result, grit plays an important role in influencing educational success in digital environments by its role in decreasing anxiety which can enhance Students’ educational success (Kosasi and Sulastri, 2021). Regarding help-seeking behaviors, Karabenick (2003) believes that grit assists learners’ cognitive coping and seeking for help, which instead indirectly increases their desperation, separation, and educational stress. Based on the prior studies, a great degree of grit may therefore be considered a defensive guard from the detrimental effects of the coronavirus pandemic on learners (Vainio and Daukantaitė, 2016). As a psychological driving force, grit triggers and guides people’s activities in online learning interactions that are associated with academic stress and act as a buffer in the first place. It is also considered an intensifier. Such research results are consistent with the previous evidence that grit contributes to reducing adverse environmental effects (Blalock et al., 2015). Indeed, some studies have explored grit as a factor that moderates the relationship between adverse external events and internal states (e.g., emotions and behaviors) (Moles et al., 2017). Given that grit can serve as a guard against negative experiences, it moderates academic stress among international students, who experience social isolation due to COVID-19. The review of the literature revealed that the learners found the classroom environment enjoyable since they found their class a source of enthusiasm, stimulation, and cooperation because of the quiet, calm, and positive learning setting that mitigates their anxiety. This can be endorsed to the arrangement between the features of FL as a teaching model.

Drawing on research conducted on grit, teachers can provide a highly motivating environment, where there is a reduced level of anxiety. In such an environment, the emphasis is put on enhancing Students’ grit. Flipped class reduces learning anxiety, raising their interactions in classroom tasks. Research has shown that effective FL implementation contributes to paving the way for the creation of a learning environment, which has proved to play a pivotal role in alleviating participants’ anxiety.

Moreover, FL led to the optimization of time allocated to class activities that would otherwise be spent on other instructional routines (e.g., the lecture). This makes it possible for learners to do more activities. FL increases the Students’ eagerness and confidence thanks to more opportunities created for them before class. Whereas the teacher retains the place of a manager and the collaboration between the learner and the teacher happens in a formal situation, the flipped classroom empowers the teacher to become the facilitator and observer and learners could be more ready to be involved and committed to the digital classroom settings. Consequently, the flipped classroom can alleviate anxiety in classes and increase their self-assurance and presentation. Moreover, based on the review of literature, the participants believed that the classroom was more attractive and this made them more motivated, inspired, and competitive due to the calm, enjoyable, and encouraging learning environment. These positive outcomes can be attributed to the consistency between the features of FL as a teaching method and the collaborative nature of active learning approaches that lead to less anxiety (Abdullah et al., 2021).

Furthermore, some studies have found the positive effect of grit on determination, and self-regulation. There is a positive correlation between grit and wellbeing and mental health, whereas there is a negative correlation between grit and stress and symptoms of depression (Kannangara et al., 2018). The positive correlation can be attributed to Student’s ability to move on after experiencing some setbacks that are sources of stress for them.

All instructors must try to prepare enjoyable and useful learning tasks by encouraging the learners to engage in FL activities. This can change the learners’ attitudes and emotions for the better, reducing learners’ cognitive load in this context, and increasing their concentration on learning contexts. Moreover, when EFL teachers are aware of Students’ personality traits, grit, and anxiety, they may be pushed to take part eagerly in FL. Teachers can play an important role in motivating learners to apply social media to reinforce their achievement.

Grit, as a vigorous variable, could be taught to learners as resilience-building anticipation of academic tension during the courses to develop their interest and perseverance and decrease their stress level. Moreover, teachers, managers, and other syllabus designers are encouraged to embed the FL in the syllabus to be taught to teacher trainers since its effectiveness in alleviating anxiety is assured.

The support of the various measures of grit and anxiety entails carrying out more investigations, particularly, empirical studies. More specifically, the impact of FL on Students’ grit and anxiety can be clarified by conducting longitudinal studies. Moreover, more studies should be carried out to find out if cultural factor influences learners’ grit. The relationship between various components of grit, and the anxiety induced by having to attend classes can be the focus of other studies. One can suspect that all EFL students would not find FL useful. As a result, prospective research needs to shed light on the possible impact of different factors (e.g., socio-economic background, age, as well as a previous online learning experience) in the flipped classroom. Since both positive and negative emotions are the target of positive psychology, future research on the investigation of FL on learners’ emotions, especially from a control-value perspective, can focus on positive emotions such as enjoyment and other negative emotions like boredom.

## Ethics statement

The studies involving human participants were reviewed and approved by the Zhongyuan University of Technology Academic Ethics Committee. The patients/participants provided their written informed consent to participate in this study.

## Author contributions

The author confirms being the sole contributor of this work and has approved it for publication.
